# Overview of exosomal non-coding RNAs in cardiovascular disease using high throughput sequencing

**DOI:** 10.1016/j.ejphar.2025.178277

**Published:** 2025-10-25

**Authors:** Mortaza Eivazi, Leila Abkhooie, Kamran Hosseini, Parnia Mobasheran, Tahereh Ebrahimi, Vahideh Tarhriz, Eric Lazartigues

**Affiliations:** a Department of Computer Science, Faculty of Mathematics, Statistics, and Computer Science, University of Tabriz, Tabriz, Iran; b Department of Medical Biotechnology, Faculty of Medicine, Lorestan University of Medical Sciences, Khorramabad, Iran; c Razi Herbal Medicines Research Center, Lorestan University of Medical Sciences, Khorramabad, Iran; d Shiraz Neuroscience Research Center (SNRC), Shiraz University of Medical Sciences, Shiraz, Iran; e Cardiovascular Center of Excellence, Louisiana State University Health Sciences Center, New Orleans, LA, USA; f Department of Pharmacology and Experimental Therapeutics, Louisiana State University Health Science Center, Louisiana, USA; g Molecular Medicine Research Center, Biomedical Institute, Tabriz University of Medical Sciences, Tabriz, Iran; h South-East Louisiana Veterans Healthcare System, New Orleans, LA, USA

**Keywords:** Non-coding RNAs, Epigenetic modulation, Biomarker, Next generation sequencing, Hypertension

## Abstract

Non-coding RNAs including miRNAs, long-ncRNAs, and circular RNAs play crucial roles in cell-to-cell communication and epigenetic regulation. They control gene expression at multiple levels, including transcriptional, post-transcriptional, and translational, thereby influencing associated signaling pathways. Encapsulated within exosomes, micro-vesicles, and apoptotic bodies, ncRNAs can be transported between cells while being protected from harsh environmental conditions. Studies have shown various expression patterns across different cell types and in various physiological and pathological states. However, the precise correlations and extent of their contribution to diseases are still under investigation. Recent studies have reported the involvement of extracellular vesicle-associated ncRNAs in the pathogenesis of cardiovascular diseases, such as hypertension. This involvement has been linked to the regulation of a range of cellular and molecular pathways, including the renin-angiotensin system. Because these exosomes can be stably detected and measured in body fluids, they hold promise as tools for both diagnosis and therapy. This review explores the potential diagnostic utility of cell-derived circulating extracellular vesicle ncRNA-based biomarkers and therapeutic approaches in cardiovascular diseases, utilizing the Next-Generation Sequencing techniques. Additionally, we address significant challenges, recent advancements, and prospects of extracellular vesicle biomarkers, with a focus on their clinical applications in the context of cardiovascular diseases and hypertension.

## 1. Introduction

According to recent statistics, cardiovascular diseases (CVD) are among the leading causes of death globally, accounting for a substantial proportion of worldwide mortality (Chong et al., 2024), By 2025, uncontrolled hypertension is projected to contribute to 10.8 million avoidable deaths annually, with CVD-related mortality expected to reach approximately 35.6 million deaths globally by 2050 (Joynt Maddox et al., 2024). Modern lifestyle factors including stress, unhealthy diets, and the rising prevalence of smoking among younger populations. Consequently, the development of innovative strategies that leverage advanced molecular techniques is crucial to enhancing the treatment of cardiac diseases and improving outcomes for patients with chronic hypertension (Joynt Maddox et al., 2024).

The fact that less than two percent of human DNA comprises coding regions of the genome supports the hypothesis that mechanisms beyond coding genes are involved in many diseases (ENCODE Project Consortium, 2012). In recent years, non-coding RNAs (ncRNAs) have emerged as crucial regulators of transcription in both healthy and disease states. Unlike coding regions, which are highly conserved across species, ncRNAs are often specific to developmental stages, tissues, and species. This specificity contributes to the complexity and diversity of the human transcriptome. These ncRNAs can be extracted from urine or circulating fluids (Olivares et al., 2020). Generally, the release of extracellular RNAs is classified into two main categories: exosomal (associated with extracellular vesicles) and non-exosomal. Exosomes are small vesicles released by cells that can carry ncRNAs, among other molecules, to distant sites in the body play important roles. They are involved in numerous physiological and pathological processes, including those associated with CVD (Ma et al., 2021). When encapsulated within extracellular vesicles (EV) like exosomes, ncRNAs are shielded from endogenous RNase activity and environmental stresses, rendering them highly stable. On the other hand, non-exosomal ncRNAs are linked with stabilizing factors like Argonaute protein (AGO) and various other RNA-binding proteins that protect non-exosomal ncRNAs from degradation under harsh conditions in plasma (Arroyo et al., 2011). Among the various types of ncRNAs microRNAs (miRNAs), long non-coding RNAs (lncRNAs), and circular RNAs (circRNAs) have garnered the most attention, with the majority of research focused on these three groups (Bayraktar et al., 2023). They have been studied for their stability in body fluids and their role in intercellular signaling, which makes them promising candidates for both biomarkers and therapeutic agents in CVD (Poller et al., 2018). Variation in the quantity and type of EV’s ncRNAs can reflect the pathological state of CVD, providing insights into disease status and progression (Ma and Zhang, 2023). Recent studies on EV’s ncRNAs have shed light on their significant role in the pathogenesis of CVD. These studies highlight the complexity of intercellular communication mediated by exosomes, demonstrating their involvement in critical CVD such as myocardial hypertrophy, ventricular and vascular remodeling, heart failure, and atherosclerosis. By emphasizing the potential of ncRNAs as both biomarkers and therapeutic agents, exosome research could significantly contribute to the diagnosis, monitoring, and treatment of CVD (Neufeldt et al., 2024; van Solingen et al., 2009; Yan et al., 2015). The current review provides an overview of three key exosomal ncRNAs namely lncRNAs, miRNAs and circRNAs identified through Next-Generation Sequencing (NGS) techniques, highlighting their potential as biomarkers and therapeutic targets in CVD, especially hypertension.

## 2. Classification and functions of non-coding RNAs

**Fig. 1.**
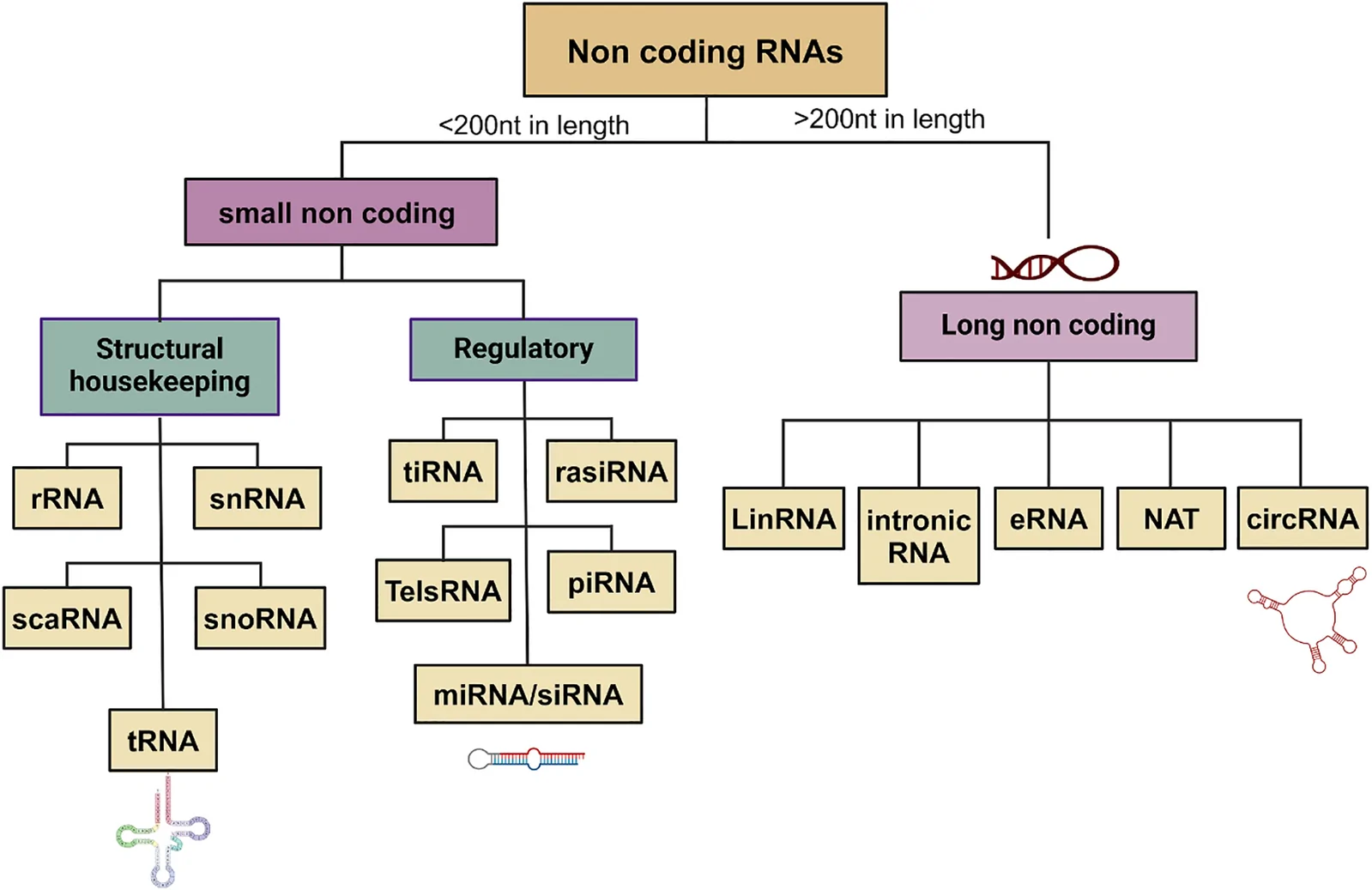
The classification of ncRNAs is based on their size, with small non-coding RNAs being less than 200 nucleotides in length and long non-coding RNAs exceeding 200 nucleotides.

**Fig. 2.**
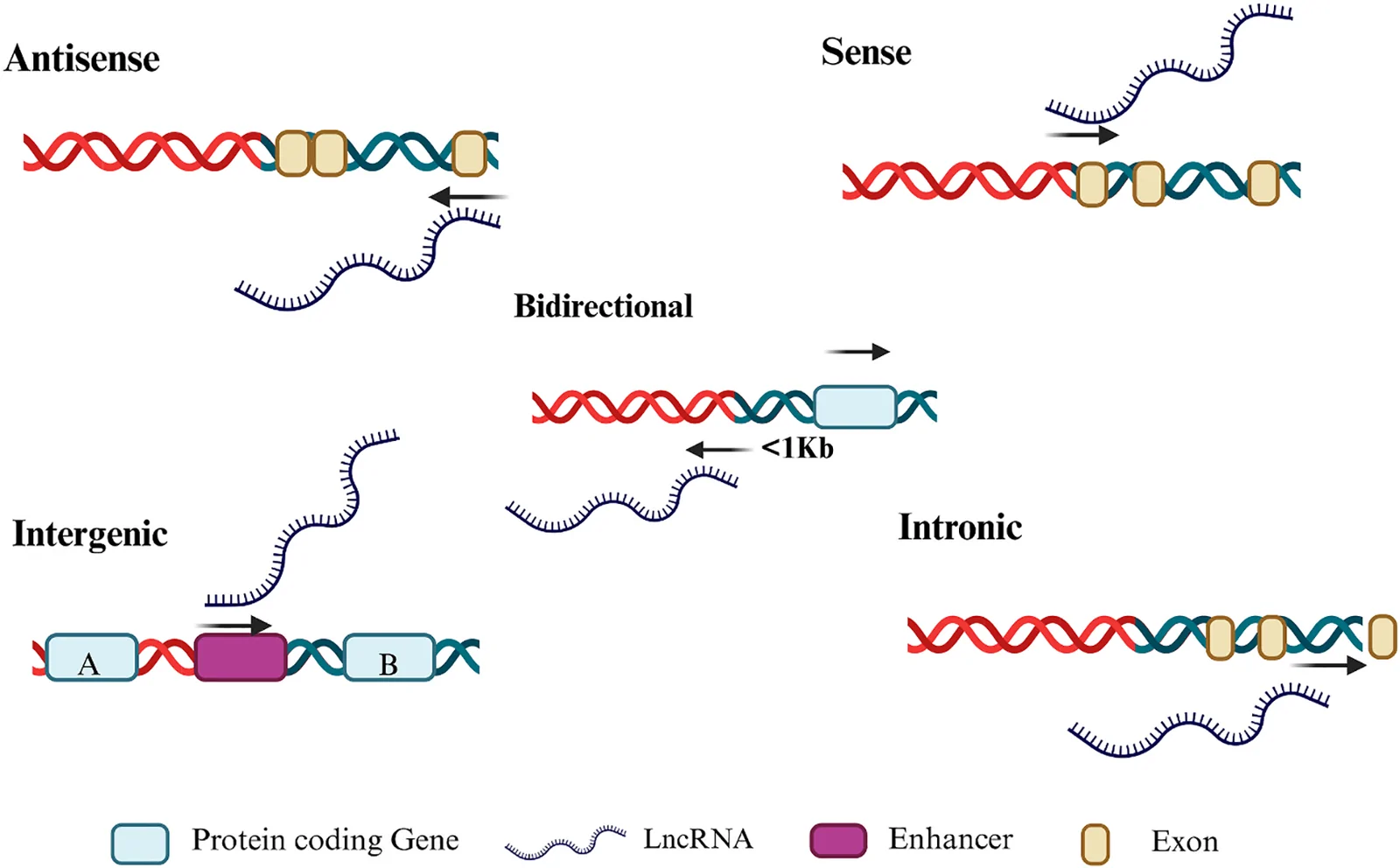
LncRNA in the context of different genomic locations. In general, there are five main categories of lncRNAs based on their location and orientation within genomic regions: a) Antisense type: lncRNAs are able to be transcribed from the opposite strand of the coding genes; b) Sense type: lncRNAs are able to be transcribed in the direction of the coding genes; c) Bidirectional type: lncRNAs are able to be transcribed in the opposite direction and in the gene promoter region of 1 kb; d) Intergenic type: lncRNAs are able to be transcribed between two coding gene regions and in the direction of the coding genes; and e) Intronic type: lncRNAs are able to be transcribed from the sense strand of the intronic region without overlapping with the exonic regions of the gene.

**Fig. 3.**
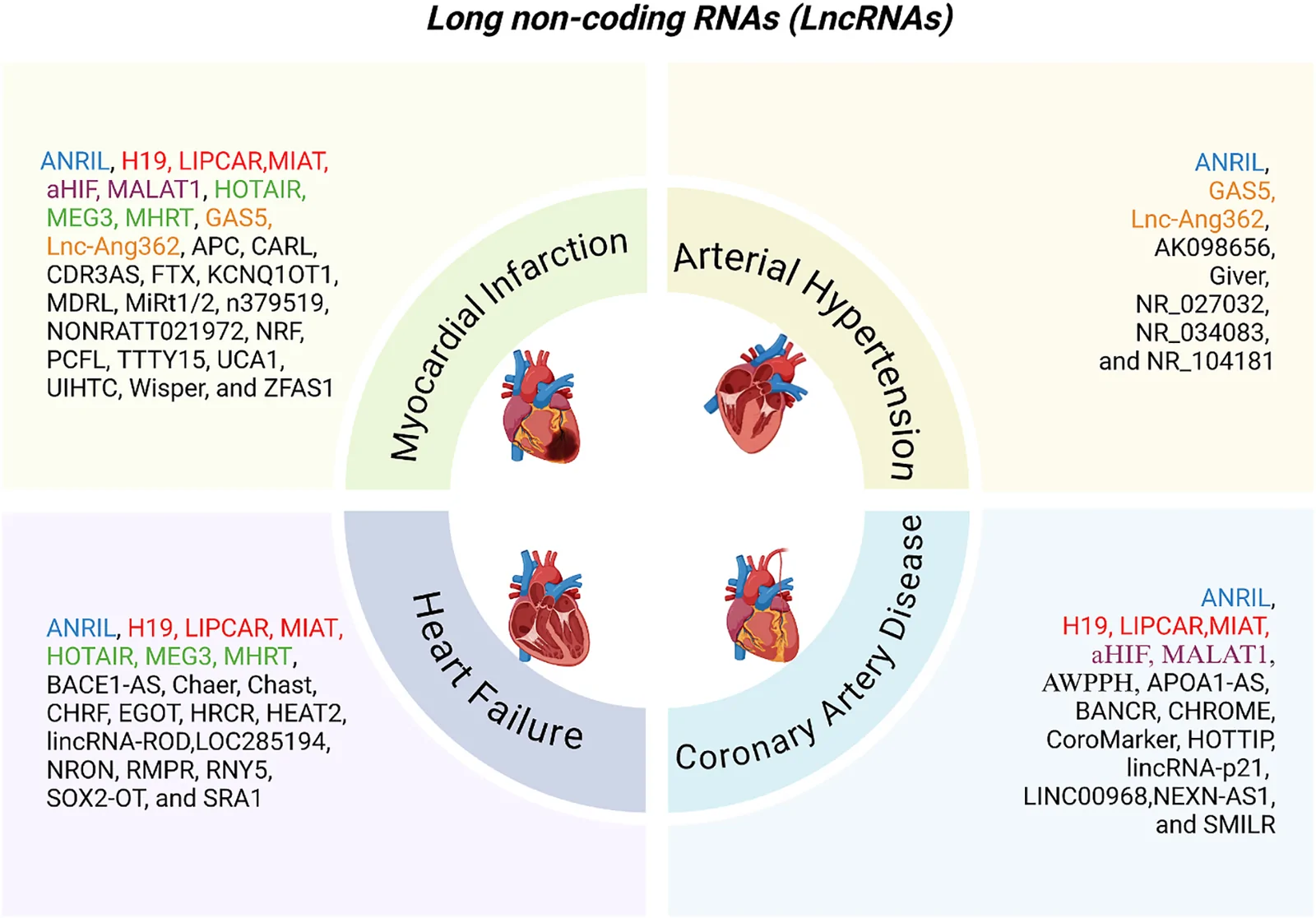
Differential expression of lncRNAs in CVDs. Blue denotes lncRNAs common to all conditions, red to myocardial infarction, coronary artery disease, and heart failure, green to myocardial infarction and heart failure, orange to myocardial infarction and hypertension, and purple to myocardial infarction and coronary artery disease.

Based on length, non-coding RNAs are divided into two groups: small non-coding RNAs (sncRNAs; shorter than 200 nucleotides) and long non-coding RNAs (lncRNAs; longer than 200 nucleotides) (Fig. 1) (Y J. Zhang et al., 2021). Recent advancements in RNA sequencing technologies have brought substantial attention to lncRNAs, which are known to play pivotal roles in various biological processes, including chromatin remodeling, epigenetic regulation, and transcriptional control (Horlacher et al., 2023; Huang et al., 2019). Fig. 2 illustrates the various types of lncRNAs based on their genomic locations and orientation relative to protein-coding genes. These categories include antisense, sense, bidirectional, intronic, and intergenic lncRNAs. Each exerting distinct regulatory functions depending on their position within or around protein-coding genes. They are produced through the transcription of genomic regions that can be exonic, intergenic, or distal to protein-coding loci (Philippe et al., 2014). They exhibit distinct features such as 3′-polyadenylation, 5′-splicing, and notable thermodynamic stability (Mattick et al., 2023). Depending on their subcellular localization, lncRNAs demonstrate functional versatility. In the nucleus, akin to microRNAs, lncRNAs regulate gene expression through modulation of chromatin architecture, transcriptional activation or repression, splicing, imprinting, and cell cycle progression (Cheng et al., 2005). LncRNAs interact with chromatin and activate transcription by recruiting transcription factors, making them essential in epigenetic gene control (Dave et al., 2023; Khalil et al., 2009). In the cytoplasm, ncRNAs interact with proteins and other RNA species to modulate mRNA translation (Abgrall et al., 1991; Gao et al., 2022; Rinn et al., 2007). Furthermore, lncRNAs have been observed to potentially serve as host transcripts for small RNAs, such as miRNAs, with both types of RNAs often being co-regulated. miRNAs regulate gene expression by binding to complementary sequences within target mRNAs, resulting in either mRNA degradation or translational inhibition. This interaction typically requires a conserved antisense sequence within the miRNA’s seed region to bind effectively to the 3′ untranslated region (3′-UTR) of the target mRNA. MiRNAs play indispensable roles in various biological processes, including development, differentiation, and the pathogenesis of diseases such as cancer and CVD (Eisen et al., 2020). Their stability and ease of detection in the circulation and other body fluids using simple methods, such as RT-PCR, have established miRNAs as potential as non-invasive biomarkers for major diseases, particularly cancer and CVD (Hunter et al., 2008; Weber et al., 2010; Zampetaki et al., 2010). Additionally, advanced techniques like microarray and NGS enable quantitative and high-resolution profiling of miRNAs in patient samples without the need for endogenous controls or pre-selected probes. These methods have emerged as gold-standard diagnostic tools, offering enhanced sensitivity, specificity, and predictive accuracy for detecting diseases at their early stages (Duan et al., 2011; Moldovan et al., 2014). Fig. 3 provides an extensive overview of the differential expressions of lncRNAs across various CVD, including arterial hypertension, myocardial infarction, coronary artery disease, and heart failure.

Recent discoveries have identified a highly stable class of non-coding RNAs known as circRNAs. These molecules regulate miRNA functions by acting as sponges, sequestering miRNAs and preventing them from interacting with their target mRNAs (M J. Li et al., 2018). CircRNAs are a unique subclass of non-coding RNAs, distinguished by their specific structure: they form a covalently closed continuous loop, lacking both 5′ and 3′ ends. This circular configuration renders them resistant to exonuclease-mediated degradation, resulting in exceptional molecular stability (Hansen et al., 2013). Additionally, circRNAs can be easily detected through the design of primers specific to their back-splice junctions, a feature unique to circRNAs and absent in other forms of non-coding RNAs. This structural hallmark enhances their utility in molecular research and diagnostics (Rybak-Wolf et al., 2015; Venø et al., 2015).

## 3. Next-Generation Sequencing for non-coding RNA biomarker discovery

Over the past two decades, numerous genes have surfaced as potential biomarkers and have become subjects of investigation for specialized drug design. Recent advances in genomics technology, including microarrays and NGS, have led to growing interest in lncRNAs in both normal cellular processes and disease progression (Khan et al., 2023). Many advances in CVD research have benefited from the rapid improvement of sequencing methods. Genome-wide association studies (GWAS) or targeted re-sequencing have made significant advancements in understanding the genetic variants influencing BP or genes related to hypertension, including single nucleotide polymorphisms (SNPs) (Walsh et al., 2023). This has raised the question of whether variants with allelic frequencies less than 1 %, such as rare mutations that are not detectable on commercial SNP arrays, could at least partly explain the observed missing heritability (Russo et al., 2018). Several BP-related loci have been located on the human genome using GWAS, including the lncRNA H19 locus, which is correlated with alterations in systolic BP (Bayoglu et al., 2016). Although various complex networks of interacting pathways regulating hypertension have been discovered (e.g., renin-angiotensin-aldosterone system (RAAS) and ENaC-related pathways), the existing efforts on rare variant analysis have not yet provided a clear answer as to where the missing heritability lies (Razavi et al., 2021). The ability to detect structural and rare variants and questions about unique variation types with phenotype can be addressed using advanced methods such as NGS (Russo et al., 2018). With the advent of NGS, it has been documented that most complex eukaryotic genomes are transcribed into ncRNAs, including lncRNAs (Charles Richard and Eichhorn, 2018) (Table 1). NGS allows for the analysis of various genes and serial analysis of gene expression (SAGE) variations to identify duplications in exons. Furthermore, NGS provides the opportunity to uncover, in a high-throughput manner, the full thorough spectrum of genomic alterations ranging from rare to common variants and from single nucleotide variants (SNVs) to insertions, deletions, and copy number variants. Discovering novel variants can provide a risk profile for the pathological processes of diseases (Li et al., 2015). NGS technology is a powerful and robust tool for elucidating a specific genetic profile of hypertensive patients, analyzing dysregulated molecular pathways, and detecting altered biological mechanisms and cellular physiology (Fazal et al., 2021). Various ncRNAs, such as Bvht and lincRNA-p21, which play crucial roles in different signaling pathways and have been linked to numerous physiological processes and diseases, have been discovered through the RNA-seq technique (Wu et al., 2018). Supplemental methods such as chromatin immunoprecipitation sequencing (ChIP-seq), RNA-immunoprecipitation sequencing (RIP-seq), and crosslinking immunoprecipitation sequencing (CLIP-seq) provide more details on how ncRNAs interact with other molecules within the cell to exert their effects. These methods expand our understanding by mapping protein-DNA/RNA interactions and chromatin/RNA modification sites (Charles Richard and Eichhorn, 2018). Alterations in gene expression provide insights for developing more precise and effective approaches to treat and diagnose diseases. For example, changes in microglial states and their associated gene expression patterns have been linked to various neurological disorders (Xu et al., 2023). Regarding, whole-exome sequencing (WES) project was established to find rare, protein-coding variants of the genome (about 2 %) associated with heart-, lung-, and blood-related diseases. Exome sequencing has been used to determine rare variants related to CVD, enabling the diagnosis of well-phenotyped cardiovascular cohorts (Marguerat et al., 2008). The next and most complete NGS approach to test the effect of rare variants is characterized by whole-genome sequencing (WGS). WES and, more so, WGS costs are still too high to analyze large sample sizes for the identification of rare variants and new mutations in hypertensive patients (Wu et al., 2019). Moreover, WES and WGS have enabled us to recognize variants in the non-coding regions of the patient’s genome, helping identify novel genes as new therapeutic targets (Koboldt et al., 2013). Currently, it is possible to perform both WGS and WES for variation detection in patients with complex disorders such as hypertension using NGS at a lower cost. Although WGS provides complete coverage compared to WES, which only covers about 1 % of the total genome, the high costs of WGS and its low depth of sequence reads have limited its widespread utilization (Nurchis et al., 2024). As sequencing costs continue to decline and the importance of lncRNAs in CVD is increasingly recognized, WGS is poised to become more cost-effective and preferable over WES in the future. WGS holds remarkable potential, particularly for personalized medicine in patients suffering from CVD. It can reveal the etiology of unknown genetic diseases and help in the diagnosis of patients with abnormal presentations of known CVD (Spielmann et al., 2022). Moreover, the integration of WGS with functional genomics techniques like RNA-seq or ChIP-seq can open novel avenues for recognizing disease-associated signaling pathways, especially those involving RAAS. This comprehensive approach allows for a deeper understanding of the genetic and environmental factors contributing to CVD and hypertension, paving the way for more precise and effective treatments (Asada et al., 2021).

## 4. EV and ncRNAs as cardiovascular biomarkers

### 4.1. Physiological roles of EV in CVD

In the cardiovascular system, EVs regulate communication under both physiological and pathological states. They mediate the shedding of enzymes, receptors, and non-coding RNAs, contributing to processes such as vascular remodeling, endothelial dysfunction, inflammation, and fibrosis (Han et al., 2022; Malik et al., 2013; Yang et al., 2018). Endothelial cell–derived EVs are elevated in atherosclerosis and serve both as markers of injury and as active participants in disease progression (Fleury et al., 2014; Rautou et al., 2012). The size of EVs also influences biodistribution and therapeutic efficiency, with smaller vesicles showing greater uptake via endocytosis compared to larger aggregates prone to degradation (de Abreu et al., 2020). Cardiomyocyte-derived EVs (CMEVs) are particularly relevant in ischemic heart disease (Han et al., 2022; Malik et al., 2013; Yang et al., 2018). Under hypoxia, they release altered cargo-including heat shock protein 60 and pro-fibrotic matrix proteins-that can exacerbate injury but also deliver adaptive signals (Han et al., 2022; Malik et al., 2013; Yang et al., 2018). For example, cardiac-specific miRNAs within CMEVs are elevated in patients with acute myocardial infarction and may orchestrate systemic repair responses by modulating bone marrow progenitor cells (Cheng et al., 2019). Similarly, hypoxia-inducible factor-1 enhances protective miRNAs (e.g., miR-126, miR-210) in endothelial EVs, suggesting therapeutic potential (Ong et al., 2014). However, the dual ability of EV cargo to promote repair or aggravate pathology underscores the need for deeper investigation into context-specific roles and therapeutic optimization.

### 4.2. Preclinical and clinical relevance of ncRNAs in the RAAS, a critical pathway in CVD

**Fig. 4.**
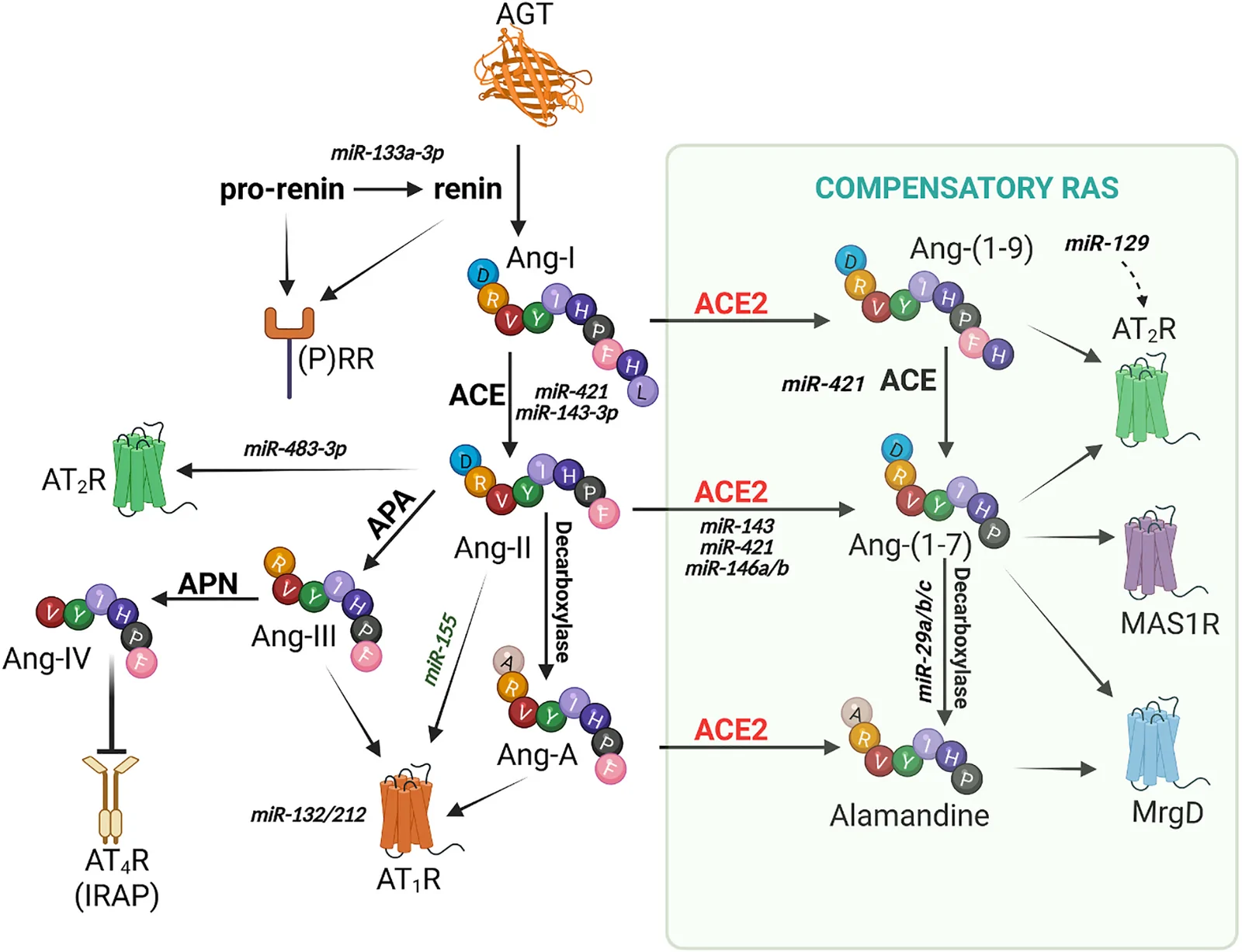
The recognized miRNAs and related targets in the RAAS.

NcRNAs exhibit distinct expression patterns that correlate with the pathophysiology of diseases, such as hypertension, making them promising candidates as therapeutic targets (Beňačka et al., 2023). Numerous studies have investigated the involvement of lncRNAs in the pathogenesis of CVD. More recently, research has increasingly focused on the direct roles of ncRNAs in regulating key RAAS-related genes. Among these, ACE stands out as a critical component of the RAAS, playing a fundamental role in blood pressure regulation. Consequently, numerous pharmaceutical inhibitors targeting ACE have been developed to treat hypertension-related pathologies (Lazartigues et al., 2007). Some studies have examined the relationship between lncRNAs and ACE. For example, miR-145 regulates the differentiation of VSMCs and vascular remodeling. MiR-145, miR-483–3p, miR-27a, and miR-27 b modulate the ACE/Ang-II/AT_1_R (Angiotensin II Type 1 Receptor) signaling pathway in hypertension by reducing ACE expression, regulating VSMCs proliferation, and cardiovascular remodeling (Chen et al., 2015; Goyal et al., 2010; Kemp et al., 2014). ACE is identified as a direct target of miR-145, and the loss of miR-143/145 in VSMCs results in the upregulation of ACE. Pharmacological inhibition of ACE using captopril effectively normalized certain molecular alterations associated with the loss of miR-143/145. The miR-143/145 cluster functions as a bicistronic unit, with both miRNAs transcribed together from a shared promoter. This bicistronic arrangement facilitates the coordinated regulation of their downstream targets. Further investigation is required to elucidate the specific mechanisms involved in the expression of miR-145 and its targeting of ACE, as miR-143 does not appear to influence ACE protein expression levels (Boettger et al., 2009; Kohlstedt et al., 2013). Moreover, bioinformatic enrichment analysis of Gene Ontology (GO) terms (https://geneontology.org/) conducted by Badi and her colleagues indicated that ACE, AT_1_R, and ACE2 could be potential targets for miR-124–3p and miR-26 b-5p (Ahmadi Badi et al., 2022). The RAAS is critically regulated by Angiotensin-Converting Enzyme 2 (ACE2), a key enzyme with significant implications for cardiovascular function and hypertension (Mohammed et al., 2020). In the brain, ACE2 modulates BP through sympathetic regulation, and recent research has highlighted advancements in ACE2-related cardiovascular studies (Mohammed et al., 2020). Xia et al. emphasized the importance of ACE2 in maintaining BP and cardiovascular homeostasis. They detailed the interactions of brain ACE2 with RAAS components, including ACE, angiotensin II (Ang II), and the AT1R, which collectively support baroreflex and autonomic function, stimulate NO release, reduce oxidative stress, and mitigate hypertension (Xia and Lazartigues, 2010). Given ACE2’s pivotal role, epigenetic research increasingly focuses on the impact of ncRNAs on ACE2, surpassing other RAAS molecules as a priority for investigation. Xu et al. identified key miRNA-lncRNA interactions involving the ACE2 gene, demonstrating that dysregulation of certain lncRNAs directly impacts ACE2 expression, contributing to the modulation of infection-related pathways. Their predictions highlighted several testis-specific lncRNAs, including GRM7-AS3, ARHGAP26-AS1, BSN-AS1, KRBOX1-AS1, CACNA1C-IT3, AC012361.1, FGF14-IT1, AC012494.1, and GS1–24F4.2, as potential interactors with ACE2. Additionally, miR-125a-5p, miR-125 b-5p, miR-574–5p, and miR-936 were highlighted as ACE2 regulators (Sabetian et al., 2021). In a clinical study involving 280 participants, a significant negative correlation was observed between lncRNA-GAS5 and miR-200 expression levels, with miR-200 directly targeting ACE2, further emphasizing the intricate regulatory network influencing ACE2 activity (Ayeldeen et al., 2024). MiR-145 reduces the phenotype transition from the contractile type to the synthetic type by reducing the expression of synthetic markers OPN, EREG, MMP2, and ADAM17-mediated ACE2 shedding in VSMCs. In addition, it activates the ACE2/Ang-(1–7)/Mas axis, leading to the structural remodeling in the metabolic hypertension (Wen et al., 2023). Studies by Satoh et al. detected that in patients with CVD and hypertension, the expression of miRNAs related to toll-like receptor 4 (TLR4), including miR-31, miR-181a, miR-16, and miR-145, decreases by the regulation of ACE2/Ang-(1–7) signaling, resulting in protective effects on patients by inhibiting the RAAS (Satoh et al., 2015). Furthermore, Apelin increased ACE2 in the failing hearts, coupled with upregulation of miR-424 and miR-503, whereas Ang-(1–7) administration enhanced cardiovascular dysfunction of Apelin-deficient mice *in vivo* (Sato et al., 2013). In diabetic nephropathy, elevated expression of miR-125 b promoted apoptosis in HK-2 cells. It seems that the increased levels of ACE2 negatively regulates miR-125 b expression, thereby mitigating its pro-apoptotic effects. By downregulating miR-125 b, ACE2 helps restore a balance in the expression of apoptosis-related genes, including Bcl-2 (anti-apoptotic) and Bax (pro-apoptotic), providing a protective effect against cell death in HK-2 cells (Huang et al., 2016). The control of miR-1, miR-133a, and miR-208 expression can occur through the ACE2/apelin pathway in cardiac hypertrophy patients treated with apelin and induces VSMC proliferation (Ceylan-Isik et al., 2013). AT_1_R and Ang II Type 2 Receptor (AT_2_R) are essential receptors in the controlling of the RAAS, regulating opposing physiological effects in cardiovascular function and hypertension. As members of the GPCR family, these angiotensin receptors play pivotal roles in blood pressure regulation, following its vasopressor role and regulation of aldosterone secretion (Ruiz-Ortega et al., 2000). Recently, Su et al. showed that a lncRNA called H19 (upregulation) through the AT_1_R by silencing let-7b (while stimulating PDGF-BB production) was able to increase the proliferation of pulmonary arterial smooth muscle cells in mice with monocrotaline-induced pulmonary arterial hypertension (PAH). Knockout of this lncRNA protected mice from pulmonary arterial remodeling and PAH following monocrotaline stimulation (Su et al., 2018). Recently, it has been identified that miR-125a/b-5p expression was directly associated with Ang II/AT_1_R signaling in mouse distal convoluted tubule cells while AT1R-associated protein) (ATRAP) was decreased. Consequently, organ-protective effects were observed with decreased miR-125–5p expression followed by increased ATRAP expression (Hirota et al., 2023). Studies by Ceolotto et al. show that a single nucleotide polymorphism (+1166C polymorphism) located in the 3′-UTR of AT_1_R can be negatively associated with miR-155 expression. In contrast, AT_1_R protein expression is positively associated with hypertension and leads to cardiovascular complications (Ceolotto et al., 2011). The miR-132/212 family has been identified as a key regulator in AT_1_R-mediated Ang II signaling, influencing vascular and cardiac remodeling in hypertension (Eskildsen et al., 2013, 2015). A reduction in the expression levels of both miR-132 and miR-212 was observed in human arteries from bypass-operated patients treated with AT_1_R blockers, suggesting that these microRNAs are involved in Ang II-induced hypertension and may be modulated by AT_1_R blockade (Eskildsen et al., 2013). Searches from various databases highlighted the interaction of lncRNAs with the Mas-related G protein-coupled receptor (MasR), which is a key component of the compensatory RAAS. Askaripour et al. conducted a study on ovariectomized rats with renal injury, demonstrating that the administration of daidzein, an isoflavonoid and agonist for GPER (G-protein estrogen receptor) along with A779 and losartan, improved renal injury and restored the expression of certain lncRNAs, including H19, MIAT, GAS5, and Rian. This effect was achieved through the modulation of MasR and AT_1_R in rats (Askaripour et al., 2023). MrgD, a Mas-related GPCR, plays a nuanced role in physiological processes such as nociception, pruritus, cell proliferation, circulation, and mast cell degranulation. It is notably expressed in sensory root ganglion neurons, the cardiovascular system, and the retina. The activation of MrgD via Alamandine is important for fine-tuning cardiovascular and sensory responses, serving as a counterbalance to AT1R activation by Ang II within the RAAS. These interactions lead to blood vessel relaxation and a subsequent decrease in BP (Suzuki et al., 2022). A search through various databases revealed no studies reporting a relationship between ncRNAs and the regulation of MrgD, (P)RR (pro-renin receptor), which primarily functions as a receptor for renin and aminopeptidases involved in the RAAS. The functional axis of miRNAs that are effective in key molecules on the RAAS, as depicted in Fig. 4.

Despite the high potential of using engineered EVs in the treatment of heart failure, there are still many technical and clinical challenges, including in the quantification techniques for EVs. These techniques include dynamic light scattering, nanoparticle tracking analysis (NTA), and resistance pulse sensing (RPS), and still have limitations in particle size and accurate discrimination (sEVs) from other physiological particles. As a result, the accuracy of detection of sample purity is low (Alvi et al., 2021; Ranjan et al., 2024). In addition, the isolation of EVs by methods such as ultracentrifugation can cause disruption of the sample transport process and for this purpose, it is better to use other methods such as ELISA to quantify the amount of EV-tagged proteins (de Freitas et al., 2021; Kwon et al., 2021). Properties including biocompatibility and immunogenicity of EVs are very important for clinical applications, and EVs of heterologous origin can stimulate immune responses (Sharma et al., 2025). Storage conditions of EVs, their stability (Qasim et al., 2019), and targeting and delivery efficiency of EVs are other critical challenges and limitations in the treatment of heart failure.

## 5. Specific ncRNA for heart failure, atherosclerosis, and hypertension

### 5.1. Heart failure

LncRNA H19 was first identified as an oncogene in some tumors. Subsequent studies have demonstrated that H19 also plays an important role in CVD, such as heart failure. Yu et al. showed that downregulation H19 expression (through TGF-β signaling pathway and targeting let-7g) leads to improvement of pulmonary vascular remodeling and right heart failure in rats with hypoxic pulmonary hypertension (HPH) by suppressing endothelial-to-mesenchymal transition (Yu et al., 2024). Li et al. investigated the relationship between H19 inhibition and improvement of right ventricular hypertrophy in the presence of sodium butyrate in heart failure patients. They showed that H19 inhibition restored the level of let-7g-5p and prevented the overexpression of IGF1 receptor and pERK in hypertrophic cardiomyocytes (M.-H M.-H. Li et al., 2024). Moreover, knockdown of H19 expression exacerbates myocardial injury by targeting CDC42 and the PI3K/AKT signaling pathway, leading to impaired cardiac remodeling in rats with heart failure (Jiao et al., 2024). MALAT1 (Metastasis Associated Lung Adenocarcinoma Transcript 1) or NEAT2, is abundantly found in cells and tissues. This lncRNA is involved in regulating the expression of genes involved in transcriptional and post-transcriptional processes. Initially, reports often associated the role of this lncRNA with cancer (Wang et al., 2017), However, it has been found that MALAT1 also plays a role in CVD and hypertension (Khan and Kirabo, 2024). In this regard, Zeng et al. showed that one of the causes of heart failure is viral myocarditis. They found that MALAT1 enhanced the enrichment of SIRT6 mRNA by UPF1 and disturbed the stability of SIRT6 mRNA to promote the development of viral myocarditis (VMC). MALAT1 can bind UPF1 to mediate SIRT6 mRNA decay and activate the Wnt/β-catenin signal pathway in VMC (Zeng et al., 2024). MicroRNA miR-21 is a prominent biomarker implicated in numerous physiological and pathological processes. It has been shown to interact with key regulatory genes, including *PTEN* (phosphatase and tensin homolog) (Akhtarkhavari et al., 2022; Zhu et al., 2007), *TPM1 (Tropomyosin 1, alpha chain)* (Akhtarkhavari et al., 2022), and *PDCD4* (Programmed Cell Death 4) (Zhu et al., 2012). Notably, miR-21 serves as a mediator of cardiovascular homeostasis and is significantly upregulated in patients with heart failure, where it interacts with BNP (J J. Zhang et al., 2017). Recent investigations have further identified that miR-21–3p, through its interaction with *CPT1A* (Carnitine Palmitoyltransferase 1 A) (Huang et al., 2024), and miR-21–5p, via its association with *ITGAV* (Integrin alpha-V) (Ranjan et al., 2024), are directly linked to the pathophysiology of heart failure. MiR-208a is encoded by the cardiac myosin heavy chain genes alpha and beta and is predominantly expressed exclusively in cardiac muscle. Studies have shown that miR-208a is crucially associated with the initiation and progression of CVD, especially heart failure (Huang et al., 2021; Matkovich et al., 2012). Hence, this miR was considered as a potential target for the prevention of heart failure (Lai et al., 2015). Gladka et al. found that in heart failure patients, function of the zinc finger E-box-binding homeobox 2 (ZEB2) protein is controlled by miR-208a. Therefore, hypertrophy of cardiac muscle cells occurs following a decrease in ZEB2 expression (Gladka et al., 2024). Montgomery et al. showed that inhibition of miR-208a resulted in decreased Myh7 expression, reduced cardiac cell remodeling, and a reversal of myosin switching in heart failure in Dahl hypertensive rats (Montgomery et al., 2011). MiR-150 is a key regulator in CVD, particularly in the pathogenesis of heart failure. Its modulation has been closely associated with cardiac remodeling and myocardial dysfunction. It has been demonstrated that miR-150 plays a pivotal role in β-adrenergic stimulation-induced cardiac remodeling in mouse models of heart failure (Russell et al., 2024). Wegiel et al. reported that miR-150–3p attenuates myocardial remodeling and exerts a protective role in heart failure (Węgiel et al., 2024). Similarly, Moukette et al. observed that miR-150 expression in cardiomyocytes from heart failure patients is upregulated through β1AR signaling mediated by β-arrestin, contributing to pathological remodeling following myocardial infarction (Moukette et al., 2022). Aonuma et al. further highlighted the role of miR-150 in targeting the *HOXA4* (Homeobox A4) gene, noting that carvedilol reduces *HOXA4* expression in cardiac fibroblasts of both humans and mice (Aonuma et al., 2022). Additionally, they identified miR-150 as a regulator of cardiac cell function in a mouse model of heart failure, where it targets *Sprr1a* (Small Proline-Rich Protein 1 A). Downregulation of *Sprr1*a in this context was shown to prevent post-myocardial infarction remodeling (Aonuma et al., 2021). These findings underscore the multifaceted role of miR-150 in cardiovascular pathology and highlight its potential as a therapeutic target in heart failure.

### 5.2. Atherosclerosis

LIPCAR (Long Intergenic Non-Protein Coding RNA, Regulator of Cardiac Remodeling) has been identified as a biomarker associated with CVD, including atherosclerosis, through its involvement in mitochondrial dysfunction, oxidative stress, and vascular inflammation (Turkieh et al., 2024). While its initial recognition was primarily linked to cardiac remodeling and heart failure (Ehgartner et al., 1987; Kumarswamy et al., 2014), LIPCAR is elevated in patients with CVD, including those with advanced atherosclerotic plaques. Therefore, it has been proposed as a biomarker for risk stratification and early identification of individuals prone to atherosclerosis-related complications (Yan et al., 2021). Antisense Non-coding RNA in the INK4 Locus (ANRIL), also known as CDKN2B-AS1, is a lncRNA that interacts with polycomb repressive complexes (PRC1 and PRC2) to mediate histone modifications, thereby upregulating pro-inflammatory cytokines and the enhancing macrophage-mediated inflammatory responses within atherosclerotic plaques (Yap et al., 2010; Zhou et al., 2016). It has been shown that dysregulation of ANRIL expression can lead to increased VSMC proliferation, contributing to plaque formation and instability. Additionally, ANRIL impairs endothelial function by inducing oxidative stress and reducing nitric oxide (NO) availability (Huang et al., 2020; Thomas et al., 2018). MEG3 (Maternally Expressed Gene 3) is a lncRNA that negatively regulates endothelial cell proliferation and promotes oxidative stress and apoptosis in endothelial cells by downregulating antioxidant pathways (Dunn-Davies et al., 2024). It has been reported that dysregulation of MEG3 levels in blood or vascular tissues is associated with atherosclerosis and may serve as a biomarker for early disease detection (Torkamandi et al., 2021). The miR-143/145 gene cluster plays a critical role in arteriolosclerosis; a condition often associated with hypertension or diabetes that leads to vessel wall thickening and luminal narrowing. In miR-143/145-deficient mice, elevated ACE (Angiotensin Converting Enzyme) levels contribute to Angiotensin (Ang) II-driven vascular tone dysregulation and the development of neointimal lesions, a hallmark of arteriosclerosis. Targeting the miR-143/145-ACE axis has therefore been proposed as a therapeutic approach for CVD, including arteriosclerosis (Boettger et al., 2009). Kontaraki et al. demonstrated that the expression levels of miR-143, miR-145, and miR-133 decreased in 60 patients with essential hypertension, while the expression levels of miR-21 and miR-1 were increased. Their findings suggest that VSMC-modulating microRNAs are closely correlated with hypertension in humans and may serve as potential therapeutic targets for the treatment of hypertension and arteriosclerosis (Kontaraki et al., 2014). Hall and colleagues investigated a single nucleotide polymorphism (SNP) rs41291957, located near the miR-143 gene, which upregulated miR-143 and miR-145, promoting a differentiated phenotype in VSMCs. This SNP appeared to be a protective genetic factor against coronary artery disease (CAD) by influencing the expression levels of miR-143/145 (Hall et al., 2021). Additionally, it was discovered that miR-143/145 were transferred from smooth muscle cells to endothelial cells through EVs. These microRNAs targeted transforming growth factor-beta (TGFβ) and controlled vascular homeostasis by downregulating target genes in endothelial cells (Climent et al., 2015). Furthermore, Elia et al. demonstrated that the absence of miR-143 and miR-145 in mice led to remarkable abnormalities in vascular structure and function, underscoring their importance in vascular health (Elia et al., 2009). Finally, Quintavalle et al. confirmed that dysregulation of the miR-143/145 cluster can contribute to vascular pathologies such as atherosclerosis and hypertension (Quintavalle et al., 2010). These studies collectively emphasized the critical role of the miR-143/145 cluster in vascular health and highlighted their involvement in CVD and atherosclerosis. MiR-33 (miR-33a and miR-33 b) regulates cholesterol and lipid metabolism and plays a crucial role in the progression of atherosclerosis by modulating lipid homeostasis, cholesterol transport, and macrophage function. MiR-33 targets ABCA1 (ATP-binding cassette transporter A1) and ABCG1, which are essential for exporting cholesterol from macrophages to high-density lipoproteins (HDL) (Rayner et al., 2010). In addition, miR-33 regulates fatty acid oxidation and lipid metabolism by targeting genes such as CPT1A (carnitine palmitoyltransferase 1 A) (Dávalos et al., 2011). MiR-33 influences macrophage polarization, promoting a pro-inflammatory phenotype (M1) while inhibiting anti-inflammatory macrophages (M2) (Ouimet et al., 2015). This shift exacerbates inflammation within atherosclerotic plaques. MiR-33 reduces the expression of genes involved in plaque stability, such as COL1A1 and COL3A1 (Z J. Chen et al., 2018), potentially increasing the risk of plaque rupture. Accordingly, anti-miR-33 oligonucleotide-based therapies are under investigation as potential interventions for atherosclerosis (Price and Fernández-Hernando, 2015). The downregulation of miR-126 is strongly associated with the development and progression of atherosclerosis. MiR-126 promotes endothelial regeneration and angiogenesis, contributing to vascular health. It has been shown that miR-126 targets VCAM-1 (vascular cell adhesion molecule 1), reducing the recruitment of inflammatory cells to the endothelium (Harris et al., 2008). Gao et al. demonstrated that miR-126 levels were reduced in patients with atherosclerosis, making it a potential biomarker for early detection and disease progression monitoring (Gao et al., 2019). Finally, miR-155 plays a key role in the progression of atherosclerotic plaques by increasing macrophage activity during inflammation. It has been shown that miR-155 enhances the production of tumor necrosis factor-alpha (TNF-α) and interleukin-6 (IL-6) by targeting SOCS1 (suppressor of cytokine signaling 1) (Migita et al., 2017). Several studies have indicated that miR-155 plays a complex role in atherosclerosis, influencing inflammation, lipid metabolism, and plaque stability, making it an appealing target for therapeutic intervention and a potential biomarker for atherosclerosis and hypertension (Nazari-Jahantigh et al., 2012; Ran et al., 2023; Tang et al., 2021).

### 5.3. Hypertension

Hypertension, or elevated BP, is a major risk factor for CVD (Kjeldsen, 2018). Epigenetic studies have highlighted the crucial role of ncRNAs in regulating gene expression linked to hypertension, which influence key pathophysiological processes underlying hypertension, including vascular remodeling, inflammation, and the regulation of the RAAS (Pandey, 2024). The expression of specific lncRNAs is directly associated with hypertension, including ANRIL, GAS5, HIF-α, H19, lncAng362, MIAT, LIPCAR, and MALAT1 (Jusic et al., 2019). Several lncRNAs, such as ANRIL, H19, LIPCAR, and Lnc-Ang362 demonstrate common expression patterns across multiple CVD, indicating their involvement in shared pathological mechanisms (Singh et al., 2023). Conversely, lncRNAs like GAS5, NEAT1, and HOTAIR are more specifically associated with arterial hypertension, highlighting their potential roles in BP regulation (Ali et al., 2023). GAS5 is broadly expressed across adult tissues and during embryonic development. Reduced expression or knockdown of GAS5 enhances endothelial cell viability and proliferation, diminishes apoptosis, and results in aberrant endothelial activation (Wang et al., 2016; Esawy et al., 2023; Tang et al., 2019). Proinflammatory cytokine-induced vascular cell-expressed RNA (Giver) has been identified as a lncRNA that can increase oxidative stress by increasing Ang II expression through the nuclear receptor NR4A3. Giver also increases the proliferation of VSMCs and controls the expression of inflammatory genes (Das et al., 2018a). Its expression is markedly elevated in patients with hypertension, and treatment with ACE inhibitors or angiotensin receptor blockers significantly reduces Giver levels (Das et al., 2017). AK098656 is significantly upregulated in hypertensive patients. Produced by VSMCs, it promotes VSMC proliferation, migration, extracellular matrix production, and degradation of contractile proteins (Jin et al., 2018). Consequently, AK098656 may contribute to the regulation of resistance arteries, promote the synthetic phenotype of VSMCs, and potentially serve as a novel biomarker for clinical diagnosis or therapeutic intervention (Ghafouri-Fard et al., 2022; Wang et al., 2022). In PAH, Tug1 has been shown to regulate pulmonary arterial smooth muscle cell (PASMC) proliferation, thereby contributing to vascular remodeling, a hallmark of PAH (Bonnet et al., 2019). Long non-coding RNA Tug1 (taurine up-regulated gene 1) plays a significant role in vascular and renal pathologies associated with hypertension. Reports show that Tug1 is highly expressed in the aortas of spontaneously hypertensive rats (SHR) and promotes VSMC proliferation and migration. Mechanistically, Tug1 functions as a molecular sponge for miR-145–5p, which inhibits fibroblast growth factor 10 (FGF10) expression, thus defining the Tug1/miR-145–5p/FGF10 axis as a crucial regulator of VSMC dysfunction in hypertension (Shi et al., 2018). Additionally, Tug1 enhances VSMC proliferation, migration, invasion, and metastasis by sponging miR-141–3p, which targets Receptor Tyrosine Kinase-like Orphan Receptor 2 (ROR2). This Tug1/-miR-141–3p/ROR2 axis further underscores its role in accelerating VSMC progression (Tang et al., 2020). Tug1 also acts as a competing endogenous RNA (ceRNA) for miR-29 b-3p, which inhibits epithelial-to-mesenchymal transition (EMT) and extracellular matrix production in the kidney via the RAAS. Furthermore, under Ang II or aldosterone stimulation, the mineralocorticoid receptor directly interacts with Tug1 and induces its expression. Upregulation of Tug1 has been observed in fibrotic human renal biopsy samples, implicating it as a key mediator of Ang II-induced renal fibrosis (J Y. Zhang et al., 2021). Moreover, Tug1 knockdown increases miR-9–5p levels, which suppress CXCR4 expression and attenuate Ang II-induced endothelial injury. These protective effects are reversed upon miR-9–5p silencing or CXCR4 overexpression, establishing the Tug1/miR-9–5p/CXCR4 axis as a pivotal regulator of endothelial dysfunction and a promising therapeutic target for CVD and hypertension (Shi et al., 2024). Analysis of mouse models revealed that exosomal miRNAs, including miR-425–5p and miR-128–3p, exhibited approximately threefold higher levels in SHR compared to Wistar Kyoto rats (WKY). Furthermore, in patients with hypertension, miR-128–3p miRNAs act to inhibit mRNA translation and exert a negative regulatory effect on these biomolecules (X B. Liu et al., 2019). miR-128–3p also regulates cellular activities including proliferation, contraction of vascular smooth muscle cells, and methylation of VSMC myosin heavy chain 11 (*Myh11*) gene through Kruppel-like factor 4 (*KLF4*) (Farina et al., 2020a). Yin et al. documented that miR-128 promotes apoptosis and inflicts damage on cardiac cells by suppressing c-Met expression in hypertensive patients (Yin et al., 2017). Farina et al. demonstrated that miR-128 targets Kruppel-like factor 4 (Klf4), which methylates myosin heavy chain 11 (Myh11), thereby preventing intimal hyperplasia in a mouse model of carotid restenosis without altering critical cardiovascular parameters (Farina et al., 2020b). In patients with essential hypertension, elevated miR-21 expression reduces endothelial nitric oxide synthase (eNOS) levels, thereby initiating atherosclerotic processes (Cengiz et al., 2015). Li et al. reported that heightened expression of miR-21 induced by cytochrome *b* encoded by the mitochondrial genome (mt-Cytb) elevates BP in humans. This increased miR-21 expression enhances the generation of reactive oxygen species in a mouse model of hypertension (Li et al., 2016). A significant complication of hypertension is kidney damage, which has been found to affect the expression profile of 29 miRNAs in exosomes of hypertensive patients with albuminuria. Among these, miR-26a promotes podocyte damage through TGF-β signaling and may serve as a diagnostic biomarker for early renal injury (Perez-Hernandez et al., 2021). Solayman et al. demonstrated that miR-19a, miR-101, and let-7e exert β-blocking effects by regulating the β1-adrenergic receptor and other genes involved in β-blocker pharmacodynamics, thereby influencing BP regulation (Solayman et al., 2019). Important miRNAs and their functions in the RAAS are summarized in Table 2.

### 5.4. Cardiomyopathy

Cardiomyopathies constitute a heterogeneous and complex group of myocardial disorders characterized by abnormalities in the structure and function of the heart muscle, frequently culminating in heart failure and sudden cardiac death. A deeper understanding of their genetic basis and the mechanism of pathological remodeling is essential for the development of effective targeted therapies. Recent studies have revealed that ncRNAs play a pivotal regulatory role in the pathogenesis and progression of cardiomyopathies, including hypertrophic cardiomyopathy, dilated cardiomyopathy, and restrictive cardiomyopathy.

For instance, upregulation of miR-29a and miR-199a-5p has been observed in patients with severe hypertrophic cardiomyopathy, while increased expression of miR-199a-3p has been reported in mild to moderate hypertrophic cardiomyopathy. These findings indicate that these miRNAs contribute to cardiac remodeling, hypertrophy, and fibrosis (Fang et al., 2015; Zalivina et al., 2023). CircRNAs, which act as endogenous miRNA sponges and regulate gene expression, are critical in cardiac physiology-including cardiomyocyte differentiation, cardiac development, and homeostasis—as well as in the pathophysiology of cardiomyopathies. Dysregulation of circRNA expression has been implicated in the onset and progression of cardiac disorders. For example, circ-CHD7 levels are elevated in dilated cardiomyopathy patients, whereas circ-DNA6JC expression is reduced (Siede et al., 2017). Similarly, circMBOAT2 and circTMEM56 are downregulated in hypertrophic cardiomyopathy patients, suggesting that circRNAs associated with cardiomyopathies may serve as novel therapeutic targets (Sonnenschein et al., 2019). LncRNAs also regulate gene expression at epigenetic, transcriptional, and post-transcriptional levels. In a rat model of dilated cardiomyopathy, increased expression of lncRNA H19 in myocardial tissue was shown to promote cardiomyocyte apoptosis and impair left ventricular structure and function (Y Y. Zhang et al., 2017). Furthermore, research employing the CRISPR-Cas9 system in a mouse model of dilated cardiomyopathy demonstrated that deficiency of the dilated cardiomyopathy suppressor transcript, a lncRNA, resulted in cardiac dysfunction, myocardial enlargement, and mitochondrial impairment, ultimately contributing to the development of dilated cardiomyopathy (Du et al., 2024).

## 6. NGS applications in cardiovascular research

NGS technologies have substantially advanced our ability to explore the transcriptome, including lncRNAs, miRNAs, and circRNAs, in CVD and hypertension. These approaches provide high-resolution insights into gene regulation, cell-type specificity, and complex signaling pathways such as the RAAS (Han et al., 2022). Despite these advances, several technical and application-related challenges remain.

A key limitation is the detection of lncRNAs, which are often expressed at low levels, exhibit poor stability in circulation, and display tissue- or cell-specific expression (Harrison et al., 2021). While databases such as NONCODE catalog (http://www.noncode.org) tens of thousands of lncRNAs, their biological roles in hypertension remain largely unclear. Single-cell sequencing (SCS) has improved resolution in studying cellular heterogeneity, yet its integration with disease-specific transcriptomic data is still in its infancy (Han et al., 2022). For miRNA research, NGS methods like small RNA sequencing provide high sensitivity and dynamic range, allowing quantification of subtle expression changes and discovery of novel variants (Winkle et al., 2021). However, issues such as sequencing biases, the need for extensive validation (e.g., by qPCR), and data complexity present obstacles to clinical translation. The detection of rare miRNA variants and the interpretation of miRNA-mRNA interactions also remain technically demanding, requiring robust bioinformatics pipelines. (Zhang et al., 2006, 2013; Motameny et al., 2010). CircRNA studies benefit from NGS due to the ability to capture low-abundance circular isoforms that traditional methods often miss (Ruan et al., 2023). Yet, challenges include distinguishing circRNAs from linear isoforms, low expression levels, and difficulties in functional annotation. These limitations hinder the translation of circRNA findings into reliable biomarkers or therapeutic tools in CVD (Neufeldt et al., 2024). Studies conducted by researchers around the world on CVD related to circRNA using the NGS approach are summarized in Table 3. Another major challenge is the volume and complexity of sequencing data. NGS produces massive datasets requiring streamlined workflows for data extraction, normalization, and analysis (Motameny et al., 2010). Technical artifacts, low signal-to-noise ratios, and difficulties in variant annotation often complicate interpretation. Moreover, standardization across laboratories is limited, which constrains reproducibility and cross-study comparisons (Russo et al., 2018).

Beyond individual RNA species, the integration of multi-omics approaches (transcriptomics, epigenomics, and proteomics) is necessary for comprehensive biomarker discovery (Lurbe and Ingelfinger, 2021). Yet, harmonizing diverse datasets remains computationally intensive and statistically challenging. Similarly, while exosomal RNA cargo holds promise for liquid biopsy applications, isolation and characterization methods are still insufficiently standardized, and the precise source of EV in circulation is often unclear (Winkle et al., 2021).

Finally, while artificial intelligence and machine learning methods are increasingly used to mine NGS datasets, their application faces challenges related to low sample sizes, population heterogeneity, and functional validation. The interpretation of gene–gene and gene-–environment interactions from sequencing data still requires experimental confirmation, and the development of physiologically relevant models is essential to translate findings into therapy (Fawzy and Marsh, 2024; Russo et al., 2018). In summary, while NGS has opened new avenues for biomarker discovery and mechanistic insights in CVD and hypertension, its clinical translation is limited by technical constraints, data complexity, and validation requirements. Addressing these limitations-through improved sequencing accuracy, standardized workflows, robust computational tools, and functional validation-will be critical to fully harness NGS for cardiovascular research and therapy.

## 7. Conclusion

EVs have gained substantial importance in current biomedical investigations for their pivotal role in elucidating the pathogenic mechanisms of diseases, facilitating the discovery of novel biomolecules, and uncovering prospective pharmaceutical agents. As mentioned earlier, empirical evidence underscores the involvement of exosomes in advancing our understanding of disease pathophysiology and the identification of potential therapeutic targets. In this review, we explored the role of EVs particularly exosomes as important mediators of intercellular communication in CVD and hypertension. Furthermore, we underscored the significance of ncRNAs in CVD, along with the critical role played by technological advancements and attentive data management in moving forward in this research field. In CVD and hypertension, current data indicate that EVs are involved in the disease’s pathophysiological processes. Exosomes derived from hypertensive patients can provide insights into the cellular state of their origin, as exosomal contents serve as reflective indicators of parent cell conditions. As underscored in this review, EVs have a significant influence over vascular and endothelial function in CVD and hypertension, mediated by their contents. This will help to better understand and utilize EVs as potential therapeutic targets in health and disease. EVs research and their unique features have greatly advanced; however, it is imperative to develop methodologies for discovering the source of EVs and, more critically, characterizing their specific contents. This will propel research to a higher level in utilizing vesicles for the identification of the molecular underpinnings and cellular origins of diverse diseases.

## Figures and Tables

**Table 1:** Hypertension-associated long noncoding RNAs (lncRNAs).

lncRNA	Up/Down-Regulation	Class	Method of Detection	Ref.
MIAT	Up	intergenic	qPCR	X X. Li et al. (2024)
HCG22	–	intergenic	genome-wide SNP genotype qPCR	Jeong et al. (2015)
MALAT1	Up & Down	intergenic	RNASeq, Microarray, qPCR	Michalik et al. (2014)
lnc-Ang362	Up	intergenic	RNA-Seq	Leung et al. (2013)
H19	Up & Down	intergenic	Discovery meta-analysis, genome-wide SNP genotype	Su et al. (2018)
GAS5	Down	intergenic	qPCR	Wang et al. (2016)
AK098656	Up	intergenic	LncRNA microarray, whole-genome microarray	Jin et al. (2018)
AK094457	Down	intergenic	Microarray assay, qPCR	Yang et al. (2015)
SMILR	Up	intergenic	RNASeq, qPCR	Spielmann et al. (2022)
TUG1	Up	intergenic	qPCR	Yang et al. (2019)
MEG3	Down	intergenic	qPCR	Sun et al. (2017)
MRAK048635-P1	Down	intergenic	qPCR	Fang et al. (2019)
lnc-Ang 383	Up	intergenic	ChIP-seq analysis, RNA-seq, qPCR	Yue et al. (2023)
HypERlnc	Up	intergenic	RNASeq, qPCR	Bischoff et al. (2017)
sONE	Down	intergenic	qPCR	Zhang et al. (2015)
CPS1-IT	Down	intronic	qPCR	Z. Zhang et al. (2019)
UCA1	Up	intronic	Microarray assay, qPCR	Zhu et al. (2019)
ANRIL	Up	antisense	qPCR	Sun et al. (2021)
SENCR	Up	antisense	RNASeq, qPCR	Bell et al. (2014)
Hoxaas3	Up	antisense	qPCR	H. Zhang et al. (2019)
CDKN2B- AS1	Up	antisense	qPCR	Bayoglu et al. (2016)
SMANTIS	Down	antisense	Microarray analysis, qPCR	Leisegang et al. (2017)
BANCR	Up	bidirectional	qPCR	Zou et al. (2017)
XR007793	Up	–	Microarray and qPCR	Wu et al. (2018)
Giver	Up	–	RNASeq, qPCR, Microarray analysis	Das et al. (2018b)
TCONS-00034812	Down	–	qPCR, Microarray analysis	Y K. Liu et al. (2018)
LnRPT	Down	–	RNASeq, qPCR	J Z. Chen et al. (2018)
NR_033766	Down	–	Microarray analysis	Gu et al. (2015)
NONHSAT073641	Up	–	RNASeq, qPCR	Josipovic et al. (2016)
AF131217.1	Up	–	qPCR	Cao et al. (2018)

**Table 2:** The important miRNAs and their strongest targets, which interact in the RAA system.

miRs	The strongest targets based on miRTarBase	The exact roles of miRs in the RAAS/hypertension	Refs
miR-1a/b-3p	HCN4, HDAC4, FOXP1, HCN2, MET, PTMA, GPD2, CORO1C, IGF1, GJA1, XPO6, TAGLN2, SERP1, LASP1, G6PD, PNP, PAX3, SRSF9, TWF1	The upregulation of miR-1 through the ACE2/apelin pathway occurred in apelin-treated cardiac hypertrophy patients.	Ceylan-Isik et al. (2013)
		MiR-1, miR-133a, and miR-26 b display distinct expression profiles in hypertensive patients and clinical indices of left ventricular hypertrophy in hypertensive patients.	Kontaraki et al. (2014)
		The plasma levels of miR-1 are enhanced in patients with hypertension.	Chen et al. (2015)
miR-16–5p	HMGA1, CDK6, CCND3, CCND1, CCNE1, NOTCH2, FGF2, ARL2, CAPRIN1, PPM1D, CHUK	miR-16 can inhibit the RAA system in patients with coronary artery disease resulting in modulation of ACE2/Ang (1–7) signaling	Satoh et al. (2015)
		The high expression of miR-16 in hypertensive rats decreases the expression of vascular endothelial growth factor (VEGF) and increases the anti-angiogenic effect.	Fernandes et al. (2012)
miR-18a-5p	ESR1, PTEN, HIF1A, SMAD4, HSF2, ATM, DICER1	Synthesize inactive Ang 1–9 from Ang 1 to produce the vasodilatory and antiproliferative molecule Ang 1–7 from Ang II	Zhang et al. (2018)
miR-19a/b-3p	PTEN, ESR1, CCND1, ATXN1, SOCS1, TGFBR2, BMPR2, KIT, TLR2, TNF	MiR-19a/b-3p family can inhibit Ang II-induced cardiac hypertrophy by PDE5A.	K Y. Liu et al. (2018)
miR-26a/b-5p	HMGA2, HMGA1, CCNE2, PTEN, EZH2, SMAD1, RB1, GSK3B, GDAP1, MTDH, NOS2	MiR-26a-5p targets the ADAM17 gene on myocardial cells in a hypoxic model.	Wen et al. (2020)
miR-27a/b	FOXO1, PHB, ZBTB10, SP1, SPRY2, ST14, MMP13, CYP1B1, PPARG, EDNRA, EYA4	The miR-27a/27 b have been shown to deter ACE expression by directly targeting the binding sites in the 3′-UTR of ACE transcripts.	Chen et al. (2015)
miR-29a,b,c	CDK6, DNMT3A, DNMT3B, COL4A2, COL4A1, MCL1, BCL2, SFRP2, DKK1, PPM1D, PIK3R1	MiR-29a, b, c has been identified as one of the Ang II-mediated miRNAs effective in hypertension.	Ying et al. (2022)
miR-122	IGF1R, SRF, RAC1, CCNG1	MiR-122–5p inhibition prevents ATII-mediated loss of autophagy and the promotion of apoptosis and oxidative stress via activating the SIRT6-ELA-ACE2 signaling.	Song et al. (2020)
miR-125a-5p	CDKN1A, TP53, VEGFA, ERBB3, ERBB2, KLF13, BAK1, MMP11	Down-regulation of miR-125a-5p expression can inhibit ATRAP and thus inhibit Ang II-AT1R signaling.	Hirota et al. (2023)
miR-125 b-5p	NKIRAS2, TP53, VDR, BAK1, ERBB3, ERBB2, LIN28A, CBFB, CYP24A1, PRDM1, TP53INP1, STAT3, E2F3, LIN28B, BBC3 PPP1CA, PRKRA, RPS6KA1, TNFAIP3, PIGF, FGFR2, MCL1, IL6R, ABTB1, HK2, E2F2, MAPK14	Down-regulation of miR-125 b-5p expression can inhibit ATRAP and thus inhibit Ang II-AT1R signaling.	Hirota et al. (2023)
		Down-regulation of miR-125 b leads to decreased ACE2 levels and mediates ROS production and high glucose-induced apoptosis.	Huang et al. (2016)
miR-126	SPRED1, PLK2, TOM1, HOXA9, CRK, VEGFA, PIK3R2, VCAM1, IRS1, SPRED1, SOX2, TWF1, TWF2, PTPN7	MiR-126 is positively associated with BP levels.	Lai et al. (2015)
miR-129	SOX4, CCP110	MiR-129 has been identified as one of the Ang II-mediated miRNAs effective in hypertension.	Lai et al. (2015)
miR-132/−212	SIRT1, CDKN1A, ARHGAP32, RB1, HBEGF	Increased levels of miR-132/−212 occurred following chronic Ang II infusion in hypertensive rats and were prevented by AT1R blocker.	Eskildsen et al. (2015)
miR-133a-3p	CDC42, HCN2, KCNQ1, KCNH2, TAGLN2, LASP1, PNP, EGFR, ARPC5, COL1A1, BCL2L1, MCL1, RFFL, IGF1R, UBA2, MMP14, ANXA2, SNX30, SGMS2	Transfection of miR-133a decreases in Ang II and AT1R levels.	Adamcova et al. (2021)
		The Ang II-induced decrease in miR-133a levels via the AMPK/ERK1/2 pathway.	Li et al. (2016)
		The upregulation of miR-133a and miR-1, along with miR-208 through the ACE2/apelin pathway, occurred in apelin-treated cardiac hypertrophy patients.	Ceylan-Isik et al. (2013)
		Ang II decreases of miR-133a. Furthermore, siPRR efficiently reduced the Ang II-induced apoptosis	B X. Liu et al. (2019)
miR-143–3p	KRAS, MYO6, DNMT3A, FNDC3B, MAPK7, HRAS, FSCN1, HK2, SERPINE1, MACC1, PTGS2, JAG1, AKT1, BCL2, SDC1, RREB1, CD44	MiR-145/miR-143 and myocardin induce the increasing expression of ACE in hypertension.	(Boettger et al., 2009; Kohlstedt et al., 2013)
		Systolic pressure by Ang II as a vasopressor stimulus in the deletion of miR-143/miR-145) does not rise.	(Elia et al., 2009; Zhan et al., 2005)
miR-145–5p	BNIP3, KLF5, SOX2, KLF4, MUC1, MYO6, CDKN1A, STAT1, YES1, IRS1, HOXA9, FSCN1, MYC, FLI1, DFFA, IFNB1, POU5F1, IGF1R, ROBO2, SRGAP1, EIF4E, CDK4, SERPINE1, VEGFA, ITGB8, SWAP70	MiR-145 regulates RAAS genes and reduces ACE-1 expression by declining miR-145 in hypertension.	Hu et al. (2014)
		MiR-143/miR-145 and myocardia induce the increasing expression of ACE in hypertension.	(Boettger et al., 2009; Kohlstedt et al., 2013)
miR-146a	CXCR4, KIT, TLR2, TRAF6, IRAK1, ROCK1, BRCA1, CCNA2, PA2G4, FAS, CD40LG	Upregulation of miR-146a expression following telmisartan treatment improves ACE2/Ang (1–7) levels and reduces vascular remodeling in hypertension.	Takahashi et al. (2010)
miR-155	MEIS1, Table 2, MECP2, SOCS1, MLH1, INPP5D, HIVEP2, ZNF652, BACH1	MiR-155 promotes Ang II-induced proliferation of VSMCs by inhibiting AT1R.	Ceolotto et al. (2011)
miR-181a-5p	BCL2, PROX1, CDKN1B, ATM, BCL2L11, HRAS, RNF2, RALA, KLF6	The over-activation of the sympathetic system causes an increase in renin secretion and thus leads to a decrease in miR-181a.	Jackson et al. (2020)
		MiR-181a down-regulates renin expression to down-regulate RAS tone.	Adamcova et al. (2021)
miR-183	FOXO1, EZR, PDCD4, AKAP12	The expression of miR-183, frontal box P1 (FOXP1), and G protein subunit gamma 7 (GNG7) has been characterized in the placental tissue of patients with Pre-eclampsia (PE).	Lai and Yu (2020)
		MiR-183 targets the TIAM1 gene and after Ang II stimulation in cardiomyocytes transfected with miR-183 mRNA and protein decreasing occurs	Gong et al. (2022)
miR-200	TUBB3, BMI1, ZEB2, CTNNB1	Down-regulation of miR-200a controls the progression of hypertension by regulating trophoblast proliferation, migration and invasion.	Wu et al. (2020)
miR-206–3p	MET, NOTCH3, ESR1, PAX3, KRAS	By increasing the expression of miR-205 and miR-206 high BP from nephropathy was reduced.	Zhang et al. (2023)
miR-361	VEGFA	The expression of miR-361–5p along with dietary history can be a suitable marker for salt-sensitive hypertension.	Ye et al. (2017)
		The level of miR-361–3p is less in the plasma of PAH patients.	Zhang et al. (2020)
miR-302	CDK4, CCND1, LEFTY1, LEFTY2 TGFBR2, RHOC NR2F2	Increasing miR-302a may raise HTR-8/SVneo cell proliferation and repress angiogenesis by targeting VEGFA and resulting can be a potential target for the development of preeclampsia therapies.	Wu et al. (2021)
miR-376a	PIK3R1, IGF1R	Decreasing miRNA-376c expression in preeclampsia (PE) patients affected the occurrence and development of PE by downregulating the expression of 25-OH-VD.	J M. Li et al. (2018)
miR-410	MDM2, MET	MiR-410 expression is increased by increased Ang II expression leading to induce robust cardiomyocyte proliferation, myocardial infarction, and cardiomyopathies related to muscular dystrophy.	Clark et al. (2016)
		Pro-hypertrophic miRNAs such as miR-410 increase hypertrophic signaling via silencing anti-hypertrophic proteins or targeting inhibitors of ERK/AKT pathways or inducing inflammation.	Adamcova et al. (2021)
		MiR-410 prevents AT1R and avoids angiogenesis through the ERK1/2 pathway.	Hassani et al. (2023)
miR-421	ATM, SMAD4	MiR-421 reduced ACE2 protein levels and the loss of this miRNA reversed these effects. MiR-421 regulated ACE2 expression by translational repression.	Chen et al. (2015)
miR-424	WEE1, CCNE1, CCND3, CDK6, CCND1, MAP2K1, FGFR1, SIAH1, HIF1A, CUL2, SPI1	Apelin increased ACE2 in the failing hearts, coupled with upregulation of miR-424 and miR-503, whereas Ang (1–7) administration enhanced cardiovascular dysfunction of Apelin-deficient mice *in vivo*.	Sato et al. (2013)
miR-483	SMAD4, BBC3, SRF, MAPK3, IGF1, CDK4, ALCAM	MiR-483 reduced ACE2 protein levels and the loss of this miRNA reversed these effects. MiR-483 regulated ACE2 expression by translational repression.	Chen et al. (2015)
miR-503	CCND1	Apelin increased ACE2 in the failing hearts, coupled with upregulation of miR-424 and miR-503, whereas Ang (1–7) administration enhanced cardiovascular dysfunction of Apelin-deficient mice *in vivo*.	Sato et al. (2013)
miR-520	CDKN1A, ABCG2, CD44, IL8, SIRT1, CD46, PFKP	An increase of miR-520 was related to a risk of later development of gestational BP.	(Shi et al., 2015; Yang et al., 2021)
		MiR-520 b inhibits endothelial cell inflammation by down-regulating NF-κB p65-ICAM1/VCAM1 axis.	

**Table 3:** Identified circRNAs via NGS: Targets, miRNA Interactions, and Mechanisms in CVDs.

Type of circRNA	circRNA	Cells, animals, patients	Targeting miRNA	Downstream genes	Gene target	Study results	Ref.
circ-Stt3b	Up	ADSCs, HL-1 cells, and mouse	miR-15a-5p	GPX4	Down	Exosomes from hypoxic ADSCs ameliorate cardiac damage post-MI through circ-Stt3b/miR-15a-5p/GPX4 signaling activation and decreased ferroptosis	J J. Liu et al. (2024)
circZFPM2	Down	Rat cardiomyocyte, hiPSC-CMs, hypertrophic cardiomyopathy patients	–	BNP and ANP	Up	CircZFPM2 silencing promotes hypertrophy, metabolic, and cytotoxic effects in neonatal primary rat cardiomyocytes; and CircZFPM2 overexpression partially reverses the hypertrophic phenotype of hypertrophic cardiomyopathy -CMs	Neufeldt et al. (2024)
circ-0001558, circ-0001535, circ-0000972	Up	AMI patients	–	–	–	Indicator for AMI diagnosis	X X. Liu et al. (2024)
circ-RCAN2 circ-C12orf29	Down	Domestic pigs	–	–	–	These circRNAs exhibited differential regulation by myocardial infarction *in vivo* and by hypoxia in vitro	Mester-Tonczar et al. (2021)
circ-Arhgap12	Up	Mouse heart tissue	miR-135a-5p	ADCY1	–	These circRNAs modulate doxorubicin-induced cardiotoxicity by sponging miR-135a-5p	Wang et al. (2021)
circ-ALMS1_6	Up	Dilated cardiomyopathy samples	miR-133a-5p miR-133a-3p	c	–	Data from this study provides a valuable resource for exploring the function of circRNAs in human heart disease and establishes a functional paradigm for identifying novel circRNAs in other tissues	K K. Dong et al. (2020)
circ-0005540	Up	CAD patiens	miR-221 and miR-145	–	–	Diagnostic biomarker of CAD	Wu et al. (2020)
circ-0097435	Up	Patients with heart failure	miR-609, miR-1294, miR-6799–5p, miR-5000–5p, miR-96–5p	AGO2	Down	Hsa_circ_0097435 overexpression promoted cardiomyocyte apoptosis, and silencing hsa_circ_0097435 inhibited apoptosis	Han et al. (2020)
circ:chr1:95609447–95616975 circ:chr15:55640530–55640923	Up	PBMCs of patients with acute ischemic stroke	let-7c-5p miR-298	Act 1	–	Diagnostic biomarkers or potential therapeutic targets for acute ischemic stroke	Z Z. Dong et al. (2020)
circ_0005360 circ-0002168	Down	Patient samples with abdominal aortic aneurysm	miR-181 b miR-15a	–	–	Identification of circRNA expressed in patients with abdominal aortic aneurysm (AAA) and regulative role in the initiation and progression of disease	Zhou et al. (2020)
circ-0008516	Up	Mice with myocardial ischemia reperfusion injury	miR-6955–5p, miR-6966–3p	Ppp2r5b	Down	Involvement of circRNA as potential new targets on AMPK signaling in myocardial ischemia reperfusion-injury	Sun et al. (2019)
circMARK3	Up	AAAD patients	miR1273g-3p	Fgr	Up	Potential biomarker for acute Stanford type A aortic dissection (AAAD) diagnosis	Tian et al. (2019)
circRNA19591, circRNA19596, circRNA16175	Down	Atrial tissues from patients	miR-29b-1-5p, miR-29b-2-5p	–	–	Involvement of circRNAs in atrial fibrillation due to rheumatic heart disease	Hu et al. (2019)
circRNA002085 circRNA001321	–	Male patients with NPAF	miR–21, miR-1	–	–	Potential biomarker for nonvalvular persistent atrial fibrillation (NPAF)	H. Zhang et al. (2019)
chr5:90817794|90827570, chr8:71336875|71337745, chr6:22033342|22038870	Up	Rat coronary artery endothelial cells	–	TGF-β1	Up	This data provides insights into the role of circRNAs in EndMT-related CVDs	Huang et al. (2018)
circSLC8A1–1	Up	Human and mouse	–	Titin (TTN), RYR2, DMD	Up	Functional investigation into the different cardiac-expressed circRNA isoforms	Tan et al. (2017)

## Data Availability

No data was used for the research described in the article.

## Abbreviations

Ang II: angiotensin II
AGO: argonaute
ANRIL: antisense lncRNA at the INK4 locus
CAD: coronary artery disease
ChIP-seq: Chromatin Immunoprecipitation Sequencing
circRNA: Circular RNAs
CLIP-seq: Crosslinking Immunoprecipitation Sequencing
CVD: cardiovascular disease
ECM: Extracellular matrix
ECs: endothelial cells
EMT: epithelial-mesenchymal transition
eNOS: endothelial Nitric Oxide Synthase
EPCs: Endothelial progenitor cells
Exo-ncRNAs: Exosomal non-coding RNAs
GAS5: Growth Arrest-Specific 5
GWAS: Genome-Wide Association Studies
lncRNA: long non coding RNA
iPSC: induced pluripotent stem cells
miRNAs: microRNAs
mRNA: messenger RNA
MS: Mass Spectrometry
MSCs: mesenchymal stem cells
ncRNAs: non-coding RNAs
NGS: Next-Generation Sequencing
NH: Neurogenic hypertension
OPC: oligodendrocyte progenitor cells
PAH: pulmonary arterial hypertension
RAAS: renin-angiotensin-aldosterone system
RBP: RNA-binding protein
RIP-seq: RNA-Immunoprecipitation Sequencing
ROS: reactive oxygen species
RVLM: Rostral ventrolateral medulla
SAGE: Serial analysis of gene expression
SCS: Single-cell sequencing
SHR: spontaneously hypertensive rats
SIH: Stress-induced hypertension
SNVs: Single Nucleotide Variants
SNPs: Single nucleotide polymorphisms
VSMC: vascular smooth muscle cell
WES: Whole Exome Sequencing
WGS: Whole Genome Sequencing
WHO: World Health Organization
